# CRISPR-Cpf1 assisted genome editing of *Corynebacterium glutamicum*

**DOI:** 10.1038/ncomms15179

**Published:** 2017-05-04

**Authors:** Yu Jiang, Fenghui Qian, Junjie Yang, Yingmiao Liu, Feng Dong, Chongmao Xu, Bingbing Sun, Biao Chen, Xiaoshu Xu, Yan Li, Renxiao Wang, Sheng Yang

**Affiliations:** 1 Key Laboratory of Synthetic Biology, Institute of Plant Physiology and Ecology, Shanghai Institutes for Biological Sciences, Chinese Academy of Sciences, Shanghai 200032, China; 2 Shanghai Research and Development Center of Industrial Biotechnology, Shanghai 201201, China; 3 Jiangsu National Synergetic Innovation Center for Advanced Materials (SICAM), Nanjing 200237, China; 4 College of Biotechnology and Pharmaceutical Engineering, Nanjing Tech University, Nanjing 211816, China; 5 School of Pharmacy, Shanghai Jiaotong University, Shanghai 200240, China; 6 State Key Laboratory of Bioorganic and Natural Products Chemistry, Shanghai Institute of Organic Chemistry, Chinese Academy of Sciences, Shanghai 200032, China

## Abstract

*Corynebacterium glutamicum* is an important industrial metabolite producer that is difficult to genetically engineer. Although the *Streptococcus pyogenes* (*Sp*) CRISPR-Cas9 system has been adapted for genome editing of multiple bacteria, it cannot be introduced into *C. glutamicum*. Here we report a *Francisella novicida* (*Fn*) CRISPR-Cpf1-based genome-editing method for *C. glutamicum*. CRISPR-Cpf1, combined with single-stranded DNA (ssDNA) recombineering, precisely introduces small changes into the bacterial genome at efficiencies of 86–100%. Large gene deletions and insertions are also obtained using an all-in-one plasmid consisting of *Fn*Cpf1, CRISPR RNA, and homologous arms. The two CRISPR-Cpf1-assisted systems enable N iterative rounds of genome editing in 3*N*+4 or 3*N*+2 days. A proof-of-concept, codon saturation mutagenesis at G149 of γ-glutamyl kinase relieves L-proline inhibition using Cpf1-assisted ssDNA recombineering. Thus, CRISPR-Cpf1-based genome editing provides a highly efficient tool for genetic engineering of *Corynebacterium* and other bacteria that cannot utilize the *Sp* CRISPR-Cas9 system.

## Abstract (web summary)

*Corynebacterium glutamicum* is an important industrial microbe, however it has proven difficult to genetically engineer using Cas9 from *Streptococcus pyogenes*. Here the authors report effective genome engineering of the bacterium using Cpf1 from *Francisella novicida*.

C*orynebacterium glutamicum*, a high-GC content, Gram-positive soil bacterium, is an important organism for the industrial production of amino acids^1^,^2^,^3^, and it has been engineered to produce a variety of compounds, including polymer subunits and biofuels^2^,^4^. Many approaches have been developed for introducing site-directed mutations into *C. glutamicum*^5^. For example, allelic exchange methods involve either introducing a selectable marker into the edited locus or a two-step process that includes a counter-selection system^6^,^7^,^8^,^9^. More recently, phage recombination proteins have been used for recombineering, a technique that promotes homologous recombination of linear DNA or oligonucleotides^10^,^11^. However, the absence of positive selection for mutations and a low recombineering efficiency often require screening of many colonies. Due to the industrial importance of *C. glutamicum*, finding a way to take advantage of a highly efficient Clustered Regularly Interspaced Short Palindromic Repeats (CRISPR) system for robust genome editing is of high priority.

The Class 2 CRISPR-Cas prokaryote immunity system has been developed successfully as a tool for genome editing and transcription regulation, most often base on the Cas9 nuclease from *Streptococcus pyogenes*^12^,^13^,^14^,^15^. Deactivated Cas9 (dCas9)-based CRISPR interference technology has been successfully applied to *C. glutamicum* for target-specific knock-down of the expression of *pgi*, *pck* and *pyk* with an efficiency up to 97% (ref. 16). However, in our hands, genome editing of *C. glutamicum* cannot be achieved by simply replacing dCas9 with Cas9 or Cas9 nickase, possibly due to toxicity in the recipient cells. Cpf1, a single-strand RNA-guided endonuclease of the class 2 CRISPR-Cas system that cleaves targeted DNA with features distinct from those of Cas9 (ref. 17), such as utilizing a T-rich protospacer-adjencent motif (PAM) and cutting in staggered ends, may serve as an alternative to Cas9 for genome editing.

In the present work, we find that CRISPR-Cpf1 systems, when applied to *C. glutamicum* and related species, generate nucleotide substitutions, insertions and gene deletions with high efficiency (Fig. 1). When combine with single-stranded (ss) DNA recombineering technology, the CRISPR-Cpf1 system enables us to achieve genomic *in situ* codon saturation mutagenesis without relying on laborious pre-construction of libraries. CRISPR-Cpf1-assisted genome editing tools for *C. glutamicum* allow us to complete each round of iterative metabolic engineering in as little as 3 days, less than half the time required for *sacB*-based allelic exchange protocols^8^.

## Results

### Selection and functionality of Cas effector proteins

To develop a Cas9-based genome editing method, three *C. glutamicum*-*Escherichia coli* shuttle plasmids were constructed: pXMJ19ts-Pncas9 carried *Sp*Cas9 with its native promoter and a kanamycin resistance cassette, pXMJ19ts-Plcas9 carried *Sp*Cas9 under the control of the constitutive promoter PlacM^6^, and pXMJ19ts-Plcas9n carried *Sp*Cas9 nickase and PlacM. Unexpectedly, none of these plasmids could generate *C. glutamicum* (ATCC13032) transformants. In contrast, pXMJ19ts-Plcpf1, carrying Cpf1 from *Francisella novicida* (*Fn*Cpf1), transformed *C. glutamicum* at a high efficiency, close to that achieved by the pXMJ19ts control (Fig. 2a). This surprising difference in the transformation efficiency between *Sp*Cas9 and *Fn*Cpf1 suggests that the expression of *Sp*Cas9 in *C. glutamicum* is toxic. However, a Basic Local Alignment Search Tool (BLAST) search indicated that the *C. glutamicum* genome lacks a typical CRISPR RNA (crRNA) sequence of *Sp*Cas9.

To confirm that *Fn*Cpf1 is functional in *C. glutamicum*, we constructed plasmid pXMJ19ts-Plcpf1-crRNAcrtYf, which carried *Fn*Cpf1, crRNA-targeting *crtYf* (*cg0718*), and a spectinomycin resistant, pXMJ19ts compatible plasmid, pJYS2_crtYf, which carried only the *crtYf* crRNA. Plasmid pXMJ19ts-Plcpf1-crRNAcrtYf was introduced into *C. glutamicum* by bacterial transformation, and pJYS2_crtYf was introduced into *C. glutamicum* harbouring pXMJ19ts-Plcpf1. The transformation efficiencies were several orders of magnitude lower than those of their respective control plasmids (Fig. 2a). Regardless of whether the *Fn*Cpf1 and crRNA modules were combined into a single plasmid or placed separately in two compatible plasmids, they efficiently cleaved the *C. glutamicum* genome at the *crtYf* locus, thereby leading to reduced transformant survival, which was a prerequisite for positive selection of genetically edited recombinants that resist *Fn*Cpf1-mediated cleavage at the targeted site(s).

### Optimization of CRISPR-Cpf1-assisted ssDNA recombineering

**Figure 1:**
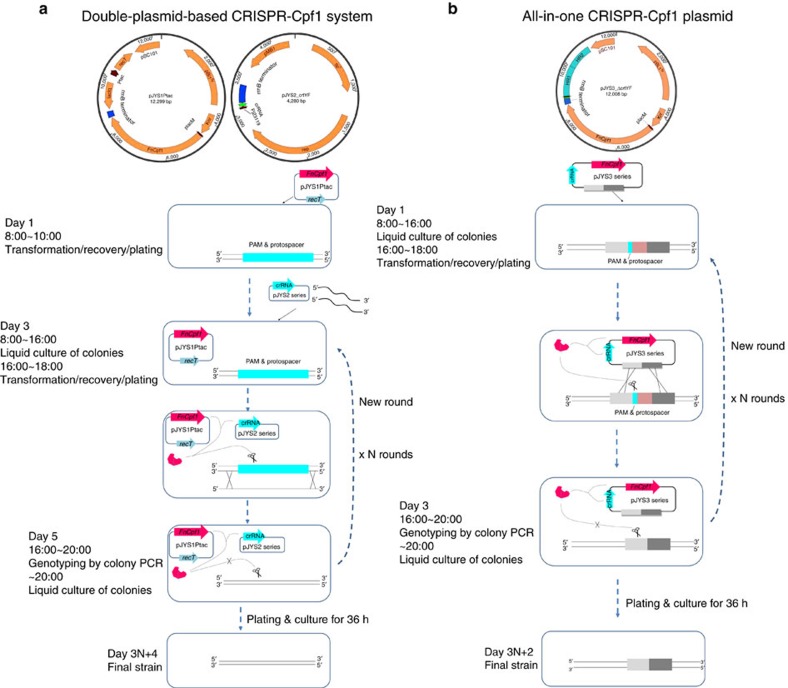
Overview of CRISPR-Cpf1-assisted genome editing in *C. glutamicum*. A flow-chart for (**a**) the double-plasmid-based CRISPR-Cpf1 system for small genome alterations, and (**b**) the all-in-one CRISPR-Cpf1 plasmid for large gene deletions and insertions. The double-plasmid-based CRISPR-Cpf1 system includes *Fn*Cpf1 and RecT expressed from pJYS1Ptac or pJYS1Peftu (Supplementary Fig. 1), respectively, and a crRNA -expressing plasmid pJYS2 series targeting different loci in the genome (indicated as *crtYf* in the figure). The pJYS2 series plasmid was co-transformed with donor ssDNA into recipient cells harbouring pJYS1 expressing RecT and *Fn*Cpf1. The pJYS2 series can be cured by overnight incubation in spectinomycin-free medium, followed by subculturing the next day for the next round of editing. The final strain was obtained by overnight culturing in antibiotic-free medium at 34 °C. The total editing time is 3*N*+4 days for *N* rounds of genome editing. The all-in-one CRISPR-Cpf1 plasmid pJYS3 series consists of *Fn*Cpf1 and crRNA, and the upstream and downstream homologous arms that served as the donor DNA. The pJYS3 series can be cured by overnight culturing in kanamycin-free medium at 34 °C, followed by subculturing the next day for the next round of editing. The total editing time is 3*N*+2 days for N rounds of genome editing. pBL1^ts^: a temperature sensitive replication derived from the pBL1 replicon of *C. glutamicum;* pSC101: a replication origin of *E. coli*; *recT*: recombination and repair protein from *E. coli*; Ptac: a *tac* promoter; Kn^r^: kanamycin resistance gene encoded by the aminoglycoside phosphotransferase gene; pMB1: a replication origin of *E. coli*; *rep*: replication origin of *C. glutamicum* derived from the pGA1 replicon; Sp^r^: spectinomycin resistance gene; Pj23119: a synthetic constitutive expression promoter; PlacM: a modified *lac* constitutive expression promoter in *C. glutamicum*; *lacIq*: *lac* repressor of *E. coli*; HR1/2: upstream/downstream homologous regions, respectively; crRNA: a matured CRISPR RNA that contains 20 bp conserved sequences and 21–24 bp targeting sequences; *Fn*Cpf1: *cpf1* derived from *F. novicida* (NC_008601).

**Figure 2:**
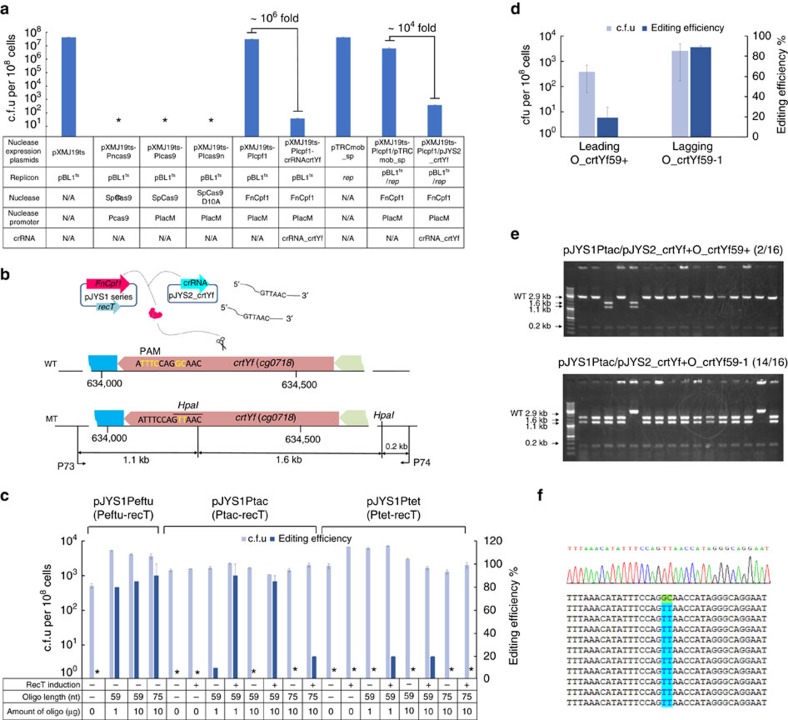
Optimization of double-plasmid-based CRISPR-Cpf1-ssDNA recombineering in *crtYf* of *C. glutamicum*. (**a**) Growth of *C. glutamicum* cells expressing the nuclease, with or without the combined expression of crRNA targeting crtYf. Asterisks indicate c.f.u. were not detected. NA, not applicable. (**b**) Overview of ssDNA-mediated nucleotide substitution in crtYf. (**c**) *C. glutamicum* cell growth and the mutation efficiency of *crtYf* after editing by 59 nt (O_crtYf59-1) or 75 bp (O_crtYf75-) template oligonucleotide targeting the lagging strand mediated by the pJYS2_crtYf and pJYS1 series carrying RecT under the control of various promoters. The mutation efficiency was determined by colony PCR, followed by HpaI digestion, as indicated in Supplementary Fig. 2. (**d**) *C. glutamicum* cell growth and the *crtYf* mutation efficiency after editing by a 59 bp lagging (O_crtYf59-1) or leading (O_crtYf59+) oligonucleotides mediated by the pJYS1Ptac/pJYS2_crtYf system. One of several parallel experiments calculating the mutation efficiency as determined by HpaI digestion is shown in **e**. (**e**) Sixteen transformants derived from lagging or leading strand targeting oligonucleotide (59 bp O_crtYf59-1 or O_crtYf59+) -mediated, pJYS1Ptac/pJYS2_crtYf -based CRISPR-Cpf1 recombineering experiments were screened by colony PCR, followed by HpaI digestion, to identify recombinants in the *crtYf* locus. A 2.9 kb fragment indicates wild-type genotype, whereas the presence of 1.6, 1.1 and 0.2 kb fragments indicate recombinant genotypes. (*n*/*N*): *n*, number of correctly edited, positive transformants; *N*, number of transformants tested. DNA ladder mix (GeneRuler, Thermo Scientific) was used as a marker. (**f**) Ten representative *crtYf* recombinants identified by HpaI digestion were further sequenced, which revealed the substitution of GC by TT for all samples, as expected. Details in transformants recording and editing efficiency calculation are listed in Supplementary Data 1. Experiments were performed in duplicates. Bar represents mean±s.d.

To develop a CRISPR-Cpf1-assisted recombineering method, three RecT-expressing plasmids based on pXMJ19ts-Plcpf1 were constructed: pJYS1Ptac, pJYS1Ptet, and pJYS1Peftu, which each express *E. coli recT* under the control of an inducible promoter, Ptac or Ptet^11^, or a constitutive promoter, Peftu^18^ (Fig. 1a; Supplementary Fig. 1). To measure editing efficiency, we designed mutations of *crtY* that would alter its protospacer seed sequence with a two-nucleotide mismatch. The mismatch was targeted by pJYS2_crtYf with the help of editing template oligonucleotide recombineering (GC to TT, and introduction of an HpaI restriction site, Fig. 2b).

Plasmid pJYS1Ptac, pJYS1Peftu, or pJYS1Ptet was introduced into *C. glutamicum*, and the resulting strains were transformed with 500 ng of pJYS2_crtYf plus various amounts of editing template (59 nucleotides or 75 oligonucleotides that target the lagging strand, O_crtYf59-1 or O_crtYf75-, respectively). Neither the length and amount of editing template nor the presence of RecT affected the recovery of transformant colony forming units (c.f.u.; Fig. 2c, Supplementary Data 1). Although the editing efficiency with *recT* transcribed from an induced Ptet was less than 20%, the fraction of edited cells increased to more than 80% if *recT* was transcribed from Peftu or induced Ptac, using 1 μg of O_crtYf59-1. Increasing the amount of O_crtYf59-1 from 1 μg to 10 μg did not significantly increase the editing efficiency when *recT* was transcribed from either Peftu or Ptac. Increasing the oligonucleotide length from 59 to 75nucleotides did not result in any significant difference in the editing efficiency (80% to 90±10%, Fig. 2c, Supplementary Data 1, Supplementary Fig. 2) using Peftu-transcribed *recT*. However, when Ptac-transcribed *recT* was used, the editing efficiency decreased by 4-fold for unknown reasons (Fig. 2c, Supplementary Data 1, Supplementary Fig. 2). There was a strong strand bias for editing efficiency: the oligonucleotide that targeted the lagging strand (O_crtYf59-) recombined four-fold more efficiently than its complementary oligonucleotide (O_crtYf59+) (Fig. 2d,e), a finding that is consistent with data obtained with *Lactobacillus reuteri*^19^,^20^. Nucleotide sequence determination of 10 HpaI-digested, correct mutants confirmed not only the presence of the introduced target mutations, but also the absence of unintended mutations in the target region (Fig. 2f). These data indicate that the CRISPR-Cpf1-assisted ssDNA recombineering system can serve as a robust and precise tool for introducing nucleotide substitutions in *C. glutamicum*.

**Figure 3:**
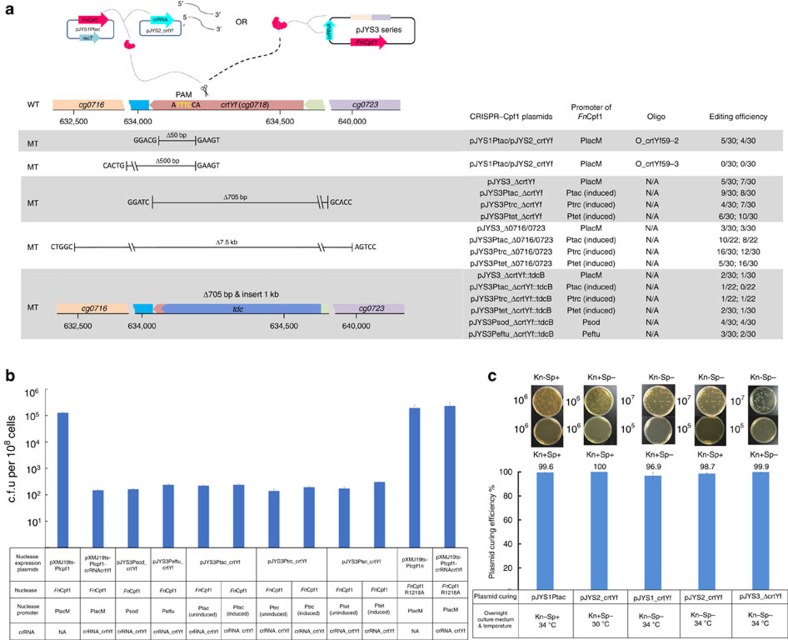
Double and all-in-one plasmid-based deletion and insertion in the *crtYf* locus of *C. glutamicum*. (**a**) Overview of *crtYf* editing by the ssDNA-mediated double-plasmid-based CRISPR-Cpf1 system (pJYS1Ptac/pJYS2_crtYf) or the all-in-one CRISPR-Cpf1 plasmid (pJYS3 series). The editing efficiency and numbers of c.f.u. of the corresponding transformants were determined by colony PCR and calculated as indicated in Supplementary Data 1 and Supplementary Fig. 3. (**b**) *C. glutamicum* growth after transformation with CRISPR-Cpf1 plasmids (pJYS3_crtYf, pJYS3Peftu_crtYf, pJYS3Psod_crtYf, pJYS3Ptac_crtYf, pJYS3Ptrc_crtYf, or pJYS3Ptet_crtYf) containing *Fn*Cpf1 under the control of various promoters (PlacM, Peftu, Psod, Ptac, Ptrc or Ptet) and the crRNA targeting *crtYf* locus. CRISPR-Cpf1 plasmid pJYS3 lacking crRNA was used as a control. The *in vivo* activity of *Fn*Cpf1 and the predicted *Fn*Cpf1 nickase R1218A were compared. Details in transformants recording and editing efficiency calculation are listed in Supplementary Data 1. (**c**) CRISPR-Cpf1 plasmid-curing efficiency for iterative genome manipulations. Cells carrying double plasmids pJYS1Ptac/pJYS2_crtYf or pJYS3_ΔcrtYf were cultured overnight in liquid BHISG with or without the indicated antibiotics at the indicated temperatures. Cultures were diluted and plated on BHISG plates with or without the indicated antibiotics and incubated for 48 h at 30 °C. The relative plasmid-curing efficiency was calculated by dividing the number of c.f.u. obtained from antibiotic-containing plates by the c.f.u. obtained from plates lacking the corresponding antibiotics. Experiments were performed in duplicates. Bar represents mean±s.d.

By using the optimized CRISPR-Cpf1-assisted ssDNA recombineering system with co-transformation of the pJYS2 plasmid series using 1 μg of the 59-nucleotide lagging-strand oligonucleotide into *C. glutamicum* harbouring pJYS1Ptac, we succeeded in obtaining a 50 bp deletion of *crtYf*; the editing efficiency was about 15% (Fig. 3a, Supplementary Data 1, Supplementary Fig. 3). However, the method failed to produce a 500 bp deletion (Fig. 3a, Supplementary Data 1, and Supplementary Fig. 3). The editing efficiency increased to 40% for the 17 bp deletion and 100% for the 2-nucleotide substitution in *argR* (*cg1585*) for the corresponding double plasmids and oligonucleotides, respectively (Supplementary Fig. 4, Supplementary Data 1). Therefore, CRISPR-Cpf1-assisted ssDNA recombineering is more suitable for small alterations than for introducing large deletions or insertions into the *C. glutamicum* genome.

### CRISPR-Cpf1-assisted gene deletion and insertion

To improve the efficiency of the CRISPR-Cpf1-assisted genome editing method for gene deletions and insertions, a set of all-in-one CRISPR-Cpf1 plasmids was constructed. Upstream and downstream homologous arms (1 kb each) were inserted into plasmid pXMJ19ts-Plcpf1-crRNAcrtYf to create pJYS3_ΔcrtYf, which aimed to delete 705 bp within *crtYf* (Fig. 1b). Transformation with 1 μg of pJYS3_ΔcrtYf into *C. glutamicum* competent cells produced more than 500 c.f.u., among which ∼15% were correctly edited (Fig. 3a, Supplementary Data 1, and Supplementary Fig. 3). Using homologous arms flanking a 7.5 kb region from *crtYf* (*cg0716*) to *cg0723*, pJYS3_Δcg0716/0723 edited *C. glutamicum* with an efficiency of 10% (Fig. 3a, Supplementary Data 1, and Supplementary Fig. 3). When a 1 kb *tdcB* gene was inserted between the homologous arms of pJYS3_ΔcrtYf, 1 μg of the resulting plasmid, pJYS3_ΔcrtYf::tdcB, when transformed into *C. glutamicum,* produced 510 c.f.u., among which ∼5% were correctly edited (Fig. 3a, Supplementary Data 1, and Supplementary Fig. 3).

Since reducing the number of CRISPR escapers (bacteria that are not cut by the Cpf1 nuclease despite of the existence of the Cpf1 cleavable target) may help improve editing efficiency, we tried to enhance Cpf1 cleavage by replacing the PlacM promoter driving *Fn*Cpf1 expression in the pJYS3 plasmid series with two strong constitutive promoters, Psod and Peftu^18^, or with three inducible promoters, Ptrc^21^, Ptac, and Ptet^11^. This construction led to the pJYS3Psod, pJYS3Peftu, pJYS3Ptac, pJYS3Ptrc and pJYS3Ptet plasmid series. Transformants were recovered with these plasmids at similar frequencies, indicating that *Fn*Cpf1 cleavage activity was similar among these constructs (Fig. 3b). The efficiencies of editing with these plasmids were also similar to those of the corresponding pJYS3 series in which *Fn*Cpf1 expression was under the control of PlacM (Fig. 3a; Supplementary Data 1; Supplementary Fig. 3). A simple explanation is that the number of edited cells was much lower than that of the CRISPR escapers, which resulted in a random pattern having a low signal-to-noise ratio regardless of varied Cpf1 expression.

In contrast to a double-strand break catalysed by native Cas9, the single-strand nick created by Cas9 nickase is prone to repair and thus increases the number of edited cells^22^. To create an *Fn*Cpf1 nickase, we prepared an R1218A mutation that corresponded to the R1226A mutant of *Acidaminococcus sp.* Cpf1, a Cpf1 variant that shows nickase activity *in vitro*^23^. However, *Fn*Cpf1R1218A did not lower transformant survival compared with the control plasmid lacking *Fn*Cpf1 (Fig. 3b). Thus, the *Fn*Cpf1 nickase cannot be used for positive selection of edited targets that tolerate *Fn*Cpf1 cleavage.

### Plasmid curing for iterative genome editing

We next cultivated edited *C. glutamicum* strains in medium supplemented with kanamycin at 30 °C to maintain the pJYS1-derivative plasmids but cure cells of the pJYS2 derivatives (they are segregationally unstable due to using a pGA1 replicon that possesses the replicative protein Rep, but not the distribution protein Per^24^). To obtain plasmid-free strains, edited *C. glutamicum* strains were then cultivated in antibiotic-free medium at 34 °C because the pJYS1 and pJYS3 derivatives are all based on the pBL1^ts^ replicon, which is temperature sensitive^25^. Loss of the corresponding plasmids was nearly 100% after an overnight incubation (Fig. 3c). This feature facilitated iterative genome editing. The operation time was 3*N*+2 or 3*N*+4 days for *N* targets with the pJYS1/pJYS2- or pJYS3-derived double or the all-in-one plasmid systems, respectively (Fig. 1).

### Applicability in other *Corynebacterium* species

**Figure 4:**
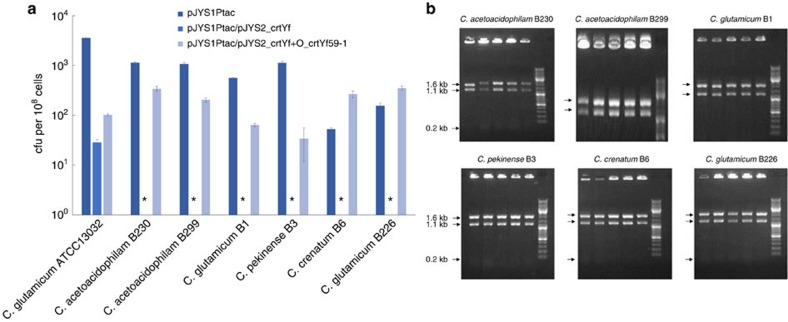
CRISPR-Cpf1-assisted recombineering in *Corynebacterium* strains. (**a**) Growth of *Corynebacterium* strains when transformed with pJYS1Ptac, the double CRISPR-Cpf1 plasmid pJYS1Ptac/pJYS2_crtYf, or pJYS1Ptac/pJYS2_crtYf, and the 59 bp oligonucleotide, O_crtYf59-1, targeting lagging strand. Stars indicate no colony was detected. Details in transformants recording and editing efficiency calculation are listed in Supplementary Data 1. Experiments were performed in duplicates. Bar represents mean±s.d. (**b**) Five transformant colonies of the indicated *Corynebacterium* strains derived from an O_crtYf59-1 -mediated, pJYS1Ptac/pJYS2_crtYf -based CRISPR-Cpf1 recombineering were analysed by colony PCR, followed by HpaI digestion, to identify recombinants in the *crtYf* locus. A 2.9 kb fragment indicates the wild-type genotype, whereas the presence of 1.6, 1.1 and 0.2 kb fragments indicate a recombinant genotype. A DNA ladder (GeneRuler) was used as a marker.

To determine whether CRISPR-Cpf1-assisted recombineering is applicable to *C. glutamicum-*related bacteria other than strain *C. glutamicum* ATCC13032, we transformed pJYS1Ptac into six industrial *Corynebacterium* strains using ATCC13032 as a control. Because electroporation-competent cells were prepared from cultures growing in tubes rather than in flasks, the overall efficiency of ATCC13032 transformation with the control plasmid pJYS1Ptac was several orders of magnitudes lower (3.56±0.05 × 10^3^ c.f.u.) (Fig. 4a; Supplementary Data 1) than observed with competent cells prepared with flask cultures (Supplementary Data 1). Nevertheless, we did not observe any obvious toxicity with the *Fn*Cpf1-expressing plasmid for the *Corynebacterium* strains tested. The resulting strains were then transformed with pJYS2_crtYf singly or together with O_crtYf59-1. The editing efficiency for a 2-nucleotide substitution was 100% for all seven *Corynebacterium* strains (Fig. 4b); the total transformant numbers varied from 34±22 to 350±40 among the various strains (Fig. 4b; Supplementary Data 1). Interestingly, except for ATCC13032, no other *Corynebacterium* strain escaped cleavage after the introduction of pJYS2_crtYf alone (Fig. 4a).

### Application in codon saturation mutagenesis

**Figure 5:**
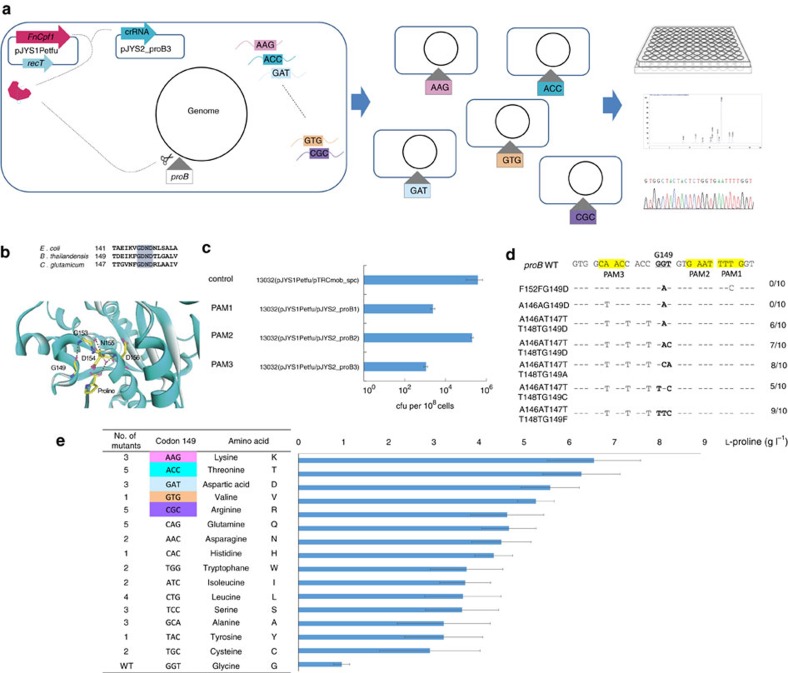
Application of CRISPR-Cpf1 system for codon saturation mutagenesis of *proB* encoding γ-glutamyl kinase. (**a**) Application of pJYS1Peftu/pJYS2_proB3 double-plasmid-based CRISPR-Cpf1 system for genomic *in situ* site-directed codon saturation of *proB* coding for γ-glutamyl kinase. A mixture of 20 different oligonucleotides was used to target codon 149 in *cg*ProB. One hundred and ninety colonies were screened by direct 96-well fermentation and high-pressure liquid chromatography (HPLC) measurement of L-proline. Codon 149 of ProB was sequenced in 42 productive mutants. (**b**) Sequence alignment of ProBs and binding mode of L-proline. The conserved residues of ProBs from *E. coli* (*ec*ProB), *B. thailandensis* (*bt*ProB), and *C. glutamicum* (*cg*ProB) are shaded. The backbone of *cg*ProB is shown in a ribbon model on which several key residues are shown in a stick model. Possible hydrogen bonds are indicated by dashed lines. (**c**) Cell growth following transformation with the pJYS1Peftu and pJYS2 series targeting the indicated three PAM regions near codon 149 of ProB as indicated in **e**. Details in transformants recording and editing efficiency calculation are listed in Supplementary Data 1. Experiments were performed in duplicates. Bar represents mean±s.d. (**d**) Effect of multiple changes on oligo recombination frequencies. Three PAM sequences near G149 of ProB are highlighted in yellow, and the wild-type codon 149 is underlined in bold. Double site-directed mutagenesis of F152FG149D and A146AG149D, and multiple mutageneses of A146AT147TT148TG149D, A146AT147TT148TG149A, A146AT147TT148TG149C, and A146AT147TT148TG149F were performed. (**e**) Forty-two *proB* recombinants with substitutions at codon 149 (table) screened by 96-well fermentation exhibited different degrees of L-proline formation (blue bars). Experiments were performed in duplicates. Bar represents mean±s.d.

The ∼100% editing efficiency of CRISPR-assisted ssDNA recombineering raised the possibility of performing codon saturation mutagenesis (mutagenesis that causes a change from a wild-type amino acid to all other amino acids) in chromosomal DNA of *C. glutamicum*. To demonstrate feasibility, we edited the γ-glutamyl kinase gene (*proB*) of *C. glutamicum* ATCC13032 to relieve feedback inhibition by L-proline (a useful chemical that is produced commercially by *C. glutamicum*^26^), thereby facilitating L-proline production (Fig. 5a).

The first and rate-limiting step in the synthesis of L-proline from L-glutamate is catalysed by γ-glutamyl kinase. With *E. coli,* this enzyme is inhibited by L-proline, and an D107N or a E143A substitution results in a loss of allosteric regulation^27^. No L-proline feedback-insensitive γ-glutamyl kinase variant from *C. glutamicum* has been reported.

A sequence alignment of ProB from *Burkholderia thailandensis* (*bt*ProB), *E. coli* (*ec*ProB), and *C. glutamicum* (*cg*ProB) indicated that D154 and N155 of *cg*ProB resemble D148 and N149 of *ec*ProB previously reported as the location for the binding motif of L-proline^28^ (Fig. 5b). Assuming that D154 and N155 of *cg*ProB are part of the active site, 3D protein structure modelling indicated that five amino-acid residues (G149, G153, D154, N155, and D156) participate in a complex hydrogen-bonding network with L-proline (Fig. 5b). Four residues (other than G149) are highly conserved among *bt*ProB, *ec*ProB, and *cg*ProB (Fig. 5b), and they are predicted to bind the substrate glutamate as determined by a previous study of D148, N149, and D150 of *ec*ProB^28^,^29^. In addition, G149 of *cg*ProB matches E143 of *ec*ProB, a site involved in L-proline inhibition^27^. Therefore, G149 was chosen as the target site for saturation mutagenesis.

To obtain a high mutation efficiency at codon G149, we first assessed the functionality of three PAMs (PAM1, TTTG; PAM2, ATTC; and PAM3, GTTG) near G149 of *proB* (Fig. 5e). Then, three corresponding pJYS2 plasmids (pJYS2-proB1, pJYS2-proB2, and pJYS2-proB3), were constructed. While pJYS2-proB2 transformed *C. glutamicum* ATCC13032 harbouring pJYS1Peftu at high efficiency (similar to the control plasmid pTrcmob_spc), the number of transformants was lower by about three orders of magnitude for pJYS2-proB1 or pJYS2-proB3 plasmids (Fig. 5d). These results are consistent with previous statistical data identifying functional PAM diversity across CRISPR-Cas systems^30^.

We next determined how many adjacent mismatches (all silent mutations) must be introduced to evade the mismatch repair system. Two oligonucleotides, F152FG149D and A146AG149D (Supplementary Data 2), were designed to construct the G149D substitution along with a silent mutation at the −2 position of PAM1 or PAM3 to inactivate PAM. No mutation was detected in 10 transformants from each of the two types. After the addition of two more silent mutations at the +1 and +3 protospacer positions of PAM3 A146AT147TT148TG149D, at least 50% of the transformant colonies were identified as mutants (Fig. 5d). These results are consistent with a previous work with *E. coli* in which changing the wobble (third) position in several nearby codons resulted in high levels of ssDNA recombination, even though the wild-type amino-acid sequence is maintained at these nearby codons^31^. Multiple substitutions of A146AT147TT148TG149A, A146AT147TT148TG149C and A146AT147TT148TG149F, which cover all substitution types at G149 of ProB, resulted in mutation efficiencies of 50–90% (Fig. 5d).

Finally, a mixture of oligonucleotides was designed for codon saturation mutagenesis of G149. We designed 20 59-oligonucleotides, each with 1 to 3 nucleotide substitutions in ProB G149, to introduce 20 codons covering 19 non-glycine amino acids and a stop codon (Supplementary Table 1). One microgram of each of the 20 oligonucleotides was mixed and co-transformed with pJYS2_proB3 into *C. glutamicum* harbouring pJYS1Peftu (Fig. 5a). Thirty transformants were analysed; 25 incorporated the desired changes, which covered 13 amino acids (Supplementary Table 2). Of the 190 colonies that were picked, 115 exhibited an L-proline titre higher than 2 g l^−1^, as compared to <1 g l^−1^ observed with wild-type ATCC13032. For 42 colonies, the L-proline titre was higher than 4 g l^−1^ (Supplementary Table 3). These mutants contained 15 amino-acid substitutions at ProB G149 (Supplementary Table 4); their effect on L-proline titres from high to low, was in the order of K>T>D>V>R>Q>N>H>W>I>L>S>A>Y>C>G (Fig. 5e). The highest L-proline titre (6.6±1.0 g l^−1^) occurred with a G149K variant.

## Discussion

CRISPR-Cas systems are now revolutionizing biotechnology, biology and medicine. So far, nearly all applications have been based on Cas9, the Cas effector protein of the type II CRISPR-Cas system. Cpf1 is a different Cas effector protein that is derived from a type V CRISPR system, which recognizes different PAM than Cas9 and cleaves DNA with a staggered rather than a flush cut. Genome editing with the CRISPR-Cpf1 endonucleases may provide an alternative to CRISPR-Cas9 endonucleases^17^, but Cpf1 has not shown any distinct advantage over Cas9. In the present work, we developed an application of Cpf1 from *F. novicida* as an efficient genome editing tool for the key industrial microorganism *C. glutamicum*. In contrast, the inherent toxicity of Cas9 from S. pyogenes to *C. glutamicum* prevented it from being used for genome editing in this bacterium.

CRISPR-Cpf1-assisted genome editing enabled the creation of nucleotide substitutions, deletions and insertions in the *C. glutamicum* chromosome. The double-plasmid system comprising the pJYS1/pJYS2 series is suitable for small changes in the genomes of *C. glutamicum* and related bacteria. N rounds of iterative genome editing can be completed in 3*N*+4 days (Fig. 1a). The ∼100% editing efficiency of CRISPR-Cpf1-assisted recombineering also enabled rapid codon saturation mutagenesis. The ability of Cpf1 to process its own crRNA can be used to simultaneously achieve multiplex genome editing using a single customized CRISPR array with *C. glutamicum*^32^.

Using the all-in-one plasmid (pJYS3 series), designed for gene insertions and deletions, N rounds of iterative genome editing can be completed in 3*N*+2 days (Fig. 1b). Both Cpf1-assisted approaches are faster than the traditional allelic exchange methods, which take 8*N* days^7^. The editing efficiency of the pJYS3 series may be further improved by increasing the homologous recombination frequency. An efficient dsDNA recombineering system is a typical way to enhance the homologous recombination by improving the efficiency of large gene deletions and insertions. However, dsDNA recombination activities associated with the Rac prophage recombinase and the exonuclease RecE/RecT from *E. coli* are rather low in *C. glutamicum*^10^. Red/ET recombination systems from native or closely related organisms need to be explored to obtain an efficient dsDNA recombineering system in *C. glutamicum*. Other Cpf1 orthologs, such as those from *Lachnospiraceae bacterium* and an *Acidaminococcus* sp, have been reported to be more efficient endonucleases in human embryonic kidney 293 cells^33^, which will allow further improvement in deletion/insertion efficiency.

To our knowledge, complete failure of Cas9 for genetic editing has not been reported previously. Because our *Sp*Cas9- or nickase-expressing plasmids, pXMJ19ts-Pncas9, pXMJ19ts-Plcas9 and pXMJ19ts-Plcas9n, lack a customized crRNA cassette, their toxicity cannot be explained by off-target cleavage. The toxicity is also unlikely to be caused by an endogenous crRNA, because we could not find any typical *Sp*Cas9 crRNA sequence in the *C. glutamicum* genome. Thus, we speculate that *Sp*Cas9 binds tightly to PAMs, even without a crRNA, thereby explaining why an inactivated *Sp*Cas9, expressed from a de-repressed Ptac promoter, produced no transformant^34^.

Given the diversity of species in which *Sp*Cas9 has been used, it is surprising that *Sp*Cas9 is toxic to *C. glutamicum* while *Fn*Cpf1 is not, although both enzymes are expressed from the same plasmid vector. One could argue that *C. glutamicum*, which belongs to Actinobacteria, a high-GC content bacterial phylum, possesses more PAM sequences for *Sp*Cas9 (NGG) than for *Fn*Cpf1 (TTTN). Interestingly, only Cas9-CRISPR interference, not Cas9-CRISPR editing, has been established in *Mycobacterium tuberculosis*, another GC-rich bacterium of the Actinobacteria phylum. Thus, *M*. *tuberculosis* may also be intolerant to *Sp*Cas9. However, *Sp*Cas9 works well in *Streptomyces* species that are also GC-rich^35^. CRISPR-Cas is exclusively derived from prokaryotes; therefore, the introduction of heterologous CRISPR-Cas components may interfere with the native CRISPR system. Clarifying differences between Cas9 and Cpf1 requires more data from other Class 2 CRISPR-Cas systems, including type V *Fn*Cpf1 and type II *Sp*Cas9 orthologs.

While addressing the problem of *Sp*Cas9 toxicity is of scientific interest, from a practical perspective, our finding that *Fn*Cpf1 behaves very differently from *Sp*Cas9 suggests that when problems of CRISPR-Cas setup are encountered, assessing different CRISPR-Cas systems, rather than fine-tuning the expression of Cas effector proteins and/or the transcription of crRNA, may be an effective solution. CRISPR nucleases having a longer PAM and different base compositions may also help reduce off-target effects.

## Methods

### Strains

All plasmids were introduced by electroporation into competent cells of *E. coli* DH5α grown in Luria–Bertani broth (LB) with ampicillin (100 mg l^−1^) at 37 °C. *C. glutamicum* strains were generally cultured in brain–heart infusion (BHI; Bacto) supplemented with 50 mM sorbitol (BHIS) unless otherwise indicated. Antibiotic concentrations, when necessary, were 25 mg l^−1^ kanamycin and 50 mg l^−1^ spectinomycin at 30 °C. Agar was added at 1.5 g l^−1^ for plates. All recombinant strains were transformed by electroporation. All strains were kept as glycerol stocks prepared in LB or BHI broth containing 20% glycerol at −80 °C. Strains and plasmids are listed in Table 1 and Supplementary Table 5.

### Plasmid constructions and oligonucleotides

All constructs used in the study are listed in Table 1 and Supplementary Table 5. Sequences of the primers, crRNAs, and oligonucleotides used in the study are in listed in Supplementary Table 5. Plasmids and chromosomal DNA were extracted using AxyPrep kits (Corning). PCRs used Taq (Thermo Scientific) or KOD-plus-neo polymerases (Toyobo). Cloning used restriction endonucleases, T4 DNA ligase (Thermo Scientific), and the isothermal assembly method^36^.

Plasmid pXMJ19ts-PnCas9 was cloned by the isothermal assembly using four DNA fragments: a temperature-sensitive replicon fragment, pBL^ts^, a kanamycin resistance gene (Kn^r^), *E. coli* replicon pSC101, and *cas9* with its native promoter. Plasmids pXMJ19ts-Plcas9, pXMJ19ts-Plcas9n, pXMJ19ts-Plcpf1, and pXMJ19ts-Plcpf1n were generated using pXMJ19-PnCas9 as a template to amplify the pSC101-pBL^ts^-Kn^r^ fragment, and they were assembled with a fragment containing various nuclease genes with a given promoter (PlacM-cas9, PlacM-cas9D10A, PlacM-cpf1 and PlacM-cpf1n, respectively) using the isothermal assembly method.

To generate pXMJ19ts-Pncas, pXMJ19 was used as a template to amplify a ∼1.5 kb fragment using primers P1/P2 and a ∼1.3 kb fragment using primers P3/P4. A temperature-sensitive replicon fragment, pBL^ts^, was obtained by overlap-extension PCR of these two fragments^25^. A ∼1.2 kb fragment of the kanamycin resistance gene (Kn^r^) was amplified using pTRCmob as a template and primers P5/P6. A ∼1.8 kb fragment of *E. coli* replicon pSC101 was amplified using pMWJ19 as a template and primers P7/P8. A ∼4.6 kb Pncas9 fragment was amplified using the pCas plasmid^37^ as a template and primers P9/P10. These four fragments were purified and recovered for isothermal assembly^36^. To generate pXMJ19ts-Plcas9, pXMJ19ts-Pncas9 was used as a template to amplify a ∼5.7 kb fragment containing pSC101-pBL^ts^-Kn^r^ using the primers P11/P12, as well as a ∼4.2 kb fragment of PlacM-*cas9* using primers P13/P14. These two fragments were purified and recovered for isothermal assembly.

To generate pXMJ19ts-Plcas9n, pXMJ19ts-Plcas9 was used as a template to amplify 5.7 and 4.2 kb fragments with primers P15/P16 and P17/P18, respectively. These two fragments were purified and recovered for isothermal assembly.

To generate pXMJ19ts-Plcpf1, pXMJ19ts-Pncas9 was used as a template to amplify a ∼5.7 kb fragment of pSC101-pBL1^ts^-Kn^r^ with primers P22/P23. A ∼3.9 kb fragment of PlacM-*Fn*Cpf1 was amplified using a synthetic *C. glutamicum* codon-optimized *Fn*Cpf1 (GenScript) as a template and primers P24/P25. These two fragments were purified and recovered for isothermal assembly. To generate pXMJ19ts-Plcpf1n, pXMJ19ts-Plcpf1 was used as a template to amplify 3.8 and 5.8 kb fragments with primers P91/P92 and P93/P94, respectively. These two fragments were purified and recovered for isothermal assembly.

To generate pXMJ19ts-Plcpf1-crRNAcrtYf and pXMJ19ts-Plcpf1n-crRNAcrtYf, pXMJ19ts-Plcpf1, pXMJ19ts-Plcpf1n were linearized with HindIII, pXMJ19 was used as a template to amplify a 295 bp fragment of the transcriptional terminator *rrnB* using primers P26/P27. Primers P28/P29 were used to amplify a 93 bp fragment of Pj23119-crRNA. These three fragments were purified and recovered for isothermal assembly.

To generate pJYS1Peftu, pXMJ19ts-Plcpf1-crRNAcrtYf was digested with SalI/XbaI to remove crRNA_crtYf to form a 9,828 bp fragment. The *C. glutamicum* ATCC13032 genome was used as a template to amplify a 361 bp Peftu promoter fragment using primers P30/P31. The *E. coli* MG1655 genome was used as a template to amplify a 924 bp *recT* fragment using primers P32/P33. These three fragments were assembled with the isothermal assembly method.

pJYS1Ptac and pJYS1Ptet were generated by replacing the *recT* promoter Peftu with Ptac or Ptet. To generate pJYS1Ptac, a 1,676 bp fragment containing Ptac and *lacIq* was amplified using primers P34/P35 and plasmid pEKEx2 as a template. A 920 bp *recT* fragment was amplified using the *E. coli* MG1655 genome as a template and primers P36/P33. pXMJ19-Plcpf1-crRNAcrtYf was digested with SwaI/XbaI to remove crRNA_crtYf (fragment 1, 9,828 bp). These three fragments were ligated with the isothermal assembly method. To generate pJYS1Ptet, primers P37/P38 and synthetic Pgap-*tetR* (GenScript) as a template were used to amplify 1,156 bp fragment 2 containing Pgap-*tetR*. Fragment 2 was used as a template, and Ptet was introduced by amplification three times: primers P37/P39 were used to amplify 1,195 bp fragment 3 of *tetR* containing a partial Ptet; primers P37/P40 and fragment 3 as a template were used to amplify 1,234 bp fragment 4 of *tetR* containing a partial Ptet; primers P37/P41 and fragment 4 as a template were used to amplify Ptet-*tetR* fragment 5 containing the complete Ptet; the *E. coli* MG1655 genome as the template and primers P42/P33 were used to amplify a 925 bp *recT* fragment (fragment 6). Fragments 1, 5 and 6 were assembled with the isothermal assembly method.

To generate pJYS2_crtYf, a 978 bp fragment containing pMB1 replicon was amplified using primers P113/P49 and plasmid pTRCmob as a template; a 386 bp fragment of crRNA targeting *crtYf* was amplified using primers P47/P48 and pXMJ19ts-Plcpf1-crRNAcrtYf as a template; a 1,996 bp fragment containing the *rep* replicon was amplified using plasmid pTRCmob as a template with primers P46/P118; a 994 bp chloramphenicol resistant gene was amplified using primers P119/P120 and plasmid pXMJ19 as a template. These four fragments were ligated and cloned via isothermal assembly. The chloramphenicol resistant gene was then replaced by Sp^r^ fragment via isothermal assemble of two fragments amplified from the above plasmid and pSenL-Spec using P121/P50 and P43/P124.

pJYS2_argR, pJYS2_proB1, pJYS2_proB2 and pJYS2_proB3 were constructed using plasmid pJYS2_crtYf as a template for whole-plasmid PCR using primers P51/P52, P53/P54, P55/P56 and P57/P58, respectively.

To generate pJYS3_ΔcrtYf, plasmid pXMJ19ts-Plcpf1-crRNAcrtYf was linearized with XbaI. The *C. glutamicum* ATCC13032 genome was used as a template to amplify 987 bp upstream and downstream homologous arms using primers P59/P60 and P61/P62, respectively. These three fragments were assembled by the isothermal assembly method. pJYS3_Δcg0716/0723 and pJYS3_ΔcrtYf::tdcB were constructed by changing the upstream and downstream homologous arms of pJYS3_ΔcrtYf. To generate pJYS3_Δcg0716/0723, pXMJ19-Plcpf1-crRNAcrtYf was linearized with XbaI to obtain fragment 7. The ATCC13032 genomic DNA was used as a template to amplify ∼1 kb upstream and downstream homologous arms using primers P63/P64 and P65/P66, respectively. These two fragments and fragment 7 were ligated with the isothermal assembly method. To generate pJYS3_ΔcrtYf::tdcB, primers P59/P67 and P68/P62 were used to amplify upstream and downstream homologous fragments, respectively, using the *C. glutamicum* ATCC13032 genome as template. The ∼1.2 kb *tdcB* fragment was amplified using the *E. coli* MG1655 genome as a template and primers P69/P70. The ∼200 bp Psod fragment was amplified using the ATCC13032 genome as a template and primers P71/P72. The Psod-*tdcB* fragment was obtained by overlap-extension PCR^25^ using the Psod and *tdcB* fragments. These three fragments and fragment 7 were assembled via the isothermal assembly method.

The PlacM promoter that drives *Fn*Cpf1 expression of the aforementioned plasmids was replaced by Ptrc, Ptac, or Ptet to generate the corresponding plasmids. To generate pJYS3Psod_ΔcrtYf::tdcB and pJYS3Peftu_ΔcrtYf::tdcB, pJYS3_ΔcrtYf::tdcB was linearized by KpnI and the Psod and Peftu fragments were amplified using *C. glutamicum* ATCC13032 genome as a template and primers P79/P80 and P81/P82, respectively. The two fragments were assembled via the isothermal assembly method. To generate pJYS3Ptrc_ΔcrtYf, pJYS3_ΔcrtYf was linearized with KpnI/XhoI to form a 10.6 kb fragment (fragment 8). P104/P105 and P106/P107 were used to amplify 1.4 kb (fragment 9) and 1.54 kb (fragment 10) fragments from pJYS3_ΔcrtYf and pTrc99A, respectively. The above three fragments were assembled via the isothermal assembly method. pJYS3Ptac_ΔcrtYf was generated by assembling fragments 8, 9, and the fragment amplified from pJYS1Ptac by p109/p110 (fragment 14) via the isothermal assembly method. pJYS3Ptet_ΔcrtYf was generated by assembling the fragment amplified from pJYS1Ptet by P111/P112 (fragment 15) with fragments 8 and 9 via the isothermal assembly method. To generate pJYS3Ptrc_Δ0716/0723, P104/P64 and p65/p108 were used to amplify fragments 11 and 12 from pJYS3_Δ0716/0723, which were assembled with fragment 10 via the isothermal assembly method. pJYS3Ptac_Δ0716/0723 was generated by assembling fragments 11, 12, and 14 via the isothermal assembly method. pJYS3Ptet_Δ0716/0723 was generated by assembling fragments 11, 12, and 15 via the isothermal assembly method. To generate pJYS3Ptrc_ΔcrtYf::tdcB, pJYS3_ΔcrtYf::tdcB was linearized with KpnI/XhoI to generate a 1.3 kb fragment (fragment 13), which was assembled with fragments 9 and 10 via the isothermal assembly method. pJYS3Ptac_ΔcrtYf::tdcB was generated by assembling fragments 9, 13, and 14 via the isothermal assembly method. pJYS3Ptet_ΔcrtYf::tdcB was generated by assembling fragment 9, 13, and 15 via the isothermal assembly method.

Members of the pJYS3 series that lack the crRNA and the homologous arms from pJYS3_ΔcrtYf derivatives with *Fn*Cpf1 under control of different promoter: pJYS3Psod_crtYf, pJYS3Peftu_crtYf, pJYS3Ptac_crtYf, pJYS3Ptrc_crtYf or pJYS3Ptet_crtYf were constructed as follows. pJYS3Psod_crtYf or pJYS3Peftu_crtYf were generated by deleting a homologous region by assembling two fragments via the isothermal assembly method by amplification using primers, P83/P80 and P79/P84, or P83/P82 and P81/P84 using pJYS3Psod_ΔcrtYf::tdcB or pJYS3Petuf_ΔcrtYf::tdcB as templates, respectively.

To generate pJYS3Ptrc_crtYf, pJYS3Ptac_crtYf, or pJYS3Ptet_crtYf lack homologous regions, 8.5 kb KpnI/XhoI linearized, 1.4 kb P104/P105 amplified fragments from pXMJts-Plcpf1-crRNAcrtYf, P106/P107, P109/P110 or P111/P112 amplified fragment from pTrc99A, pJYS1Ptac or pJYS1Ptet were isothermally assembled.

### Preparation of cells for recombineering and electroporation

Electrocompetent *C. glutamicum* was prepared as described^38^ with modifications. *C. glutamicum* and its derivatives (*C. glutamicum* harbouring pJYS1 series) were grown on BHIS agar supplemented with 10 g l^−1^ glucose (BHISG) in the absence or presence of kanamycin (BHISG-kn, for *C. glutamicum* harbouring pJYS1 series). A single colony from each strain was inoculated into 4 ml of BHISG or BHISG-kn, and grown overnight at 30 °C with shaking at 220 r.p.m. From this pre-culture, 500 μl or 1 ml was inoculated into 50 ml of BHISG or BHISG-kn supplemented with 1 ml l^−1^ Tween 80 and 4 g l^−1^ glycine. Cultures containing pJYS1Ptac or pJYS1Ptet were supplemented with 0.5 mM isopropyl β-D-1-thiogalactopyranoside or 250 ng l^−1^ anhydrotetracycline for RecT recombinase induction. Cultures were harvested and made electrocompetent when the optical density at 600 nm (OD_600_) reached ∼1.0 (transformation efficiency decreased significantly when OD_600_ exceed 1.2). Cells were chilled on ice for 20 min and collected by centrifugation at 2,600*g* and 4 °C for 10 min and washed twice with 50 ml of 10% glycerol. Competent cells were resuspended in 500 μl of 10% glycerol, and 90 μl aliquots were stored at −80 °C. pJYS1 series should be retransformed into *C. glutamicum* to prepare competent cells in case the ssDNA-mediated editing efficiency decreased.

Before electroporation, plasmid-free *C. glutamicum* cells or those carrying the pJYS1 plasmid series were thawed on ice, mixed with 5 μl (∼500 ng) of the pJYS2 series, and 5 μl (1 to 10 μg) of ssDNA or 10 μl of the pJYS3 series (∼1 μg), and then transferred into 4 °C pre-cooled electroporation cuvettes. Electroporation was performed at 25 μF, 200 Ω and 2.5 kV. Cells were immediately transferred to 900 μl of prewarmed BHISG medium and heat-shocked for 6 min at 46 °C. The cells were grown to recover for 1–2 h at 30 °C with shaking at 170 r.p.m. Cells were then plated on BHISG containing kanamycin and spectinomycin (BHISG-kn-sp), or BHISG-kn, and incubated for 2 days for c.f.u. determinations. c.f.u. was normalized for per 10^8^ input viable cells.

For cleavage activity measurement of Cas, 100 ng pXMJ19ts, pXMJ19ts-Pncas9, pXMJ19ts-Plcas9, pXMJ19ts-Plcas9n, pXMJ19ts-Plcpf1, pXMJ19ts-Plcpf1-crRNAcrtYf, pXMJ19ts-Plcpf1n, pXMJ19ts-Plcpf1n-crRNAcrtYf and pJYS1 series, 500 ng pTRCmob_sp and pJYS2 series, and 1 μg pJYS3 series were added to one aliquot of electrocompetent cells. As a negative control, an oligonucleotide with no sequence similarity to the *C. glutamicum* genome or ddH_2_O was added to one aliquot of the electrocompetent cells to determine competence and transformation efficiency.

### Identification of edited genes

To identify positive clones with substitutions at the 276 and 277 positions of the *crtYf* open reading frame, colony PCR was conducted using primers P73/P74. PCR products were digested with HpaI. Clones with deletions in *crtYf* gene were identified by PCR using primers P75/P76 for 50 bp deletion, P73/P74 for 500 bp, 705 bp deletions and for a *tdcB* insertion in the *crtYf* locus. Clones with 7.5 kb deletions in *crtYf* were identified by PCR fragment size using primers P77/P78.

### Curing plasmids for continuous genome engineering

Recombinant *C. glutamicum* containing plasmids of the pJYS1 and pJYS2 series were incubated overnight at 30 °C in BHIS-kn or at 34 °C in BHIS containing spectinomycin (BHIS-sp). Cultures were diluted by 10^4^, 10^5^, 10^6^ and 10^7^-fold with sterile water and spread onto BHIS-kn, BHIS-sp or BHIS-kn-sp, and incubated at 30 °C for 48 h for c.f.u. determination. The loss rate for pJYS1 or pJYS2 series was calculated by dividing c.f.u. from the BHIS-kn-sp plates by that from the plates containing a single antibiotic.

Recombinant *C. glutamicum* cells containing the pJYS1 and pJYS2 series plasmids were incubated overnight at 34 °C in BHISG liquid medium and spread onto BHISG, BHISG-kn, BHISG-sp, or BHISG-kn-sp plates and incubated at 30 °C for 48 h. Simultaneous loss rate of the pJYS1 and pJYS2 series was calculated by dividing c.f.u. numbers from the BHISG-kn or BHISG-sp plates by those from the antibiotic-free plates.

When using the double-plasmid-based CRISPR-Cpf1 system for iterative genome manipulation, BHISG-kn was used for overnight cultures at 30 °C and for subcultures the next day for the next round of operation. After completing the operations, cultures were incubated overnight at 34 °C in BHISG and spread onto BHISG plates to obtain bacteria in which the pJYS1 and pJYS2 series were both lost.

When using the all-in-one CRISPR-Cpf1 plasmid pJYS3 series for continuous genome manipulation, curing of the pJYS3 series was performed in the same manner as curing the pJYS1 series described above.

### Sequence alignment and molecular modelling

To determine whether ATCC13032 has an internal CRISPR sequence, the genome sequence of ATCC13032 (NC_006958) was uploaded to CRISPRs finder online server (http://crispr.i2bc.paris-saclay.fr/Server/)^39^. An alignment was conducted using the BLAST at the National Center for Biotechnology Information.

The three-dimensional structural model of *cg*ProB was generated through homology modelling using the SWISS-MODEL server^40^,^41^,^42^ (https://swissmodel.expasy.org/). The available structure of *bt*ProB (PDB code: 4q1t, which shares 38% sequence similarity to *cg*ProB, was used as the template. Then, the binding mode of proline to *cg*ProB was predicted by molecular docking using Schrodinger software (Schrodinger LLC). The *cg*ProB protein structure was prepared using the Protein Preparation module^43^ to set the protonation state of the residues around the desired binding pocket. The proline molecule was sketched and optimized using Maestro^44^, on which the secondary amine group was protonated to bear a positive charge. A cubic grid required by docking was generated by centering at the centroid of D154 and N155 with a length of 1 nm for each dimension. Then, the proline molecule was docked into the binding site using the GLIDE module with GlideScore (v 5.0)^45^,^46^,^47^ as the scoring function. The standard precision mode of the scoring function was used to rank the docking poses. The all-atom optimized potentials for liquid simulations force field was applied to molecular docking. Other parameters were set to the default values.

### L-Proline producer construction and cultivation assay

Electrocompetent cells of *C. glutamicum* ATCC13032 containing pJYS1Peftu were prepared as described above. For *in vivo* site-directed saturation mutagenesis of ProB G149, a mixture of 20 oligonucleotides (1 μg each of 59 bp oligonucleotides with 20 μg total per mixture) was used for electroporation. Transformants were inoculated into 96-well plates for L-proline fermentation tests. Fermentation volume was 600 μl, incubation temperature was 30 °C, rotation speed was 290 r.p.m. (Innova 43, Eppendorf, Germany), and incubation time was 72 h. Proline fermentation medium was glucose·H_2_O 100 g l^−1^, corn steep liquor (Meihua Group) 20 g l^−1^, (NH_4_)_2_SO_4_ 30 g l^−1^, MgSO_4_·7H_2_O 0.4 g l^−1^, KH_2_PO_4_ 1.2 g l^−1^, urea 2 g l^−1^, CaCO_3_ 30 g l^−1^, pH7.2. Strains producing L-proline were chosen for *proB* nucleotide sequence determination.

To quantify L-proline, high-pressure liquid chromatography was performed using an Agilent 1200 series equipped with an Eclipse Plus C18, 3.5 μm, 4.6 × 100 mm, and a diode array detector (DAD G1321A) via *O*-phthalaldehyde and 9-fluorenylmethyl chloroformate derivative reaction according to the high-speed amino-acid analysis on 1.8 μm reversed-phase columns instruction provided by Agilent. The mobile phase was composed of a gradient of 4 different eluents, and the flow rate was set as 1.0 ml min^−1^ at 40 °C. Glucose was measured by high-pressure liquid chromatography (HPLC) according to the guidelines for use and care of Aminex Resin-Based Columns (Bio-Rad) using an Agilent 1200 series equipped with an Aminex HPX-87H, 7.8 × 300 mm (Bio-Rad), and a refractive index detector (RID G1362A) at 60 °C with mobile phase composed of 5 mM H_2_SO_4_ and flow rate of 1.0 ml min^−1^.

### Data availability

The authors declare that all the data supporting the findings of this study are available within the paper and its Supplementary Information files or are available from the corresponding author on request.

## Additional information

**How to cite this article:** Jiang, Y. *et al*. CRISPR-Cpf1 assisted genome editing of *Corynebacterium glutamicum*. *Nat. Commun.* **8**, 15179 doi: 10.1038/ncomms15179 (2017).

**Publisher's note:** Springer Nature remains neutral with regard to jurisdictional claims in published maps and institutional affiliations.

## Supplementary Material

Supplementary Information Supplementary figures, supplementary tables and supplementary references.

Supplementary data 1 Overview of transformation and gene editing efficiency via CRISPR-Cpf1 system in 23 *Corynebacterium strains*.

Supplementary data 2 Oligonucleotides and crRNA sequences.

## Figures and Tables

**Table 1:** List of the main strains and plasmids in this study†.

**Strains and plasmids**	**Characteristics**	**Source/reference**
*Strain*		
* C. glutamicum* ATCC13032	Type strain	American Type Culture Collection (ATCC)
*Double-plasmid-based CRISPR-Cpf1 system: Cpf1 expression plasmid*		
* *pJYS1Peftu	pBL1^ts^ *oriV*_*C.glu.*_ Kn^r^ pSC101 *oriV*_*E. coli*_ PlacM-*Fn*Cpf1 Peftu-RecT	This study (Addgene: 85546)
* *pJYS1Ptac	pBL1^ts^ *oriV*_*C.glu.*_ Kn^r^ pSC101 *oriV*_*E. coli*_ PlacM-*Fn*Cpf1 Ptac-RecT *lacIq*	This study (Addgene: 85545)
*Double-plasmid-based CRISPR-Cpf1 system: crRNA expression plasmid*		
* *pJYS2_crtYf	*rep oriV*_*C.glu.*_ Sp^r^ pMB1 *oriV*_*E. coli*_ Pj23119-crRNA targeting *crtYf*	This study (Addgene: 85544)
*All-in-one CRISPR-Cpf1 plasmid*		
* *pJYS3_ΔcrtYf	pBL1^ts^ *oriV*_*C.glu.*_ Kn^r^ pSC101 *oriV*_*E. coli*_ PlacM-*Fn*Cpf1, Pj23119-crRNA targeting *crtYf*, 1 kb upstream and downstream homologous arms flaking the 705 bp deletion fragment inside *crtYf*	This study (Addgene: 85542)

^*^A complete list of strains and plasmids is presented in Supplementary Data 2.
