# Supplementary material for: CRISPR-Cpf1 assisted genome editing of Corynebacterium glutamicum
Source: Nat Commun. 2017 May 4;8:15179. doi: 10.1038/ncomms15179 (PMC5418603; doi:10.1038/ncomms15179)
Supplement: Supplementary Information — Supplementary figures, supplementary tables and supplementary references. [file ncomms15179-s1.pdf]

Supplementary Information

Supplementary Figures

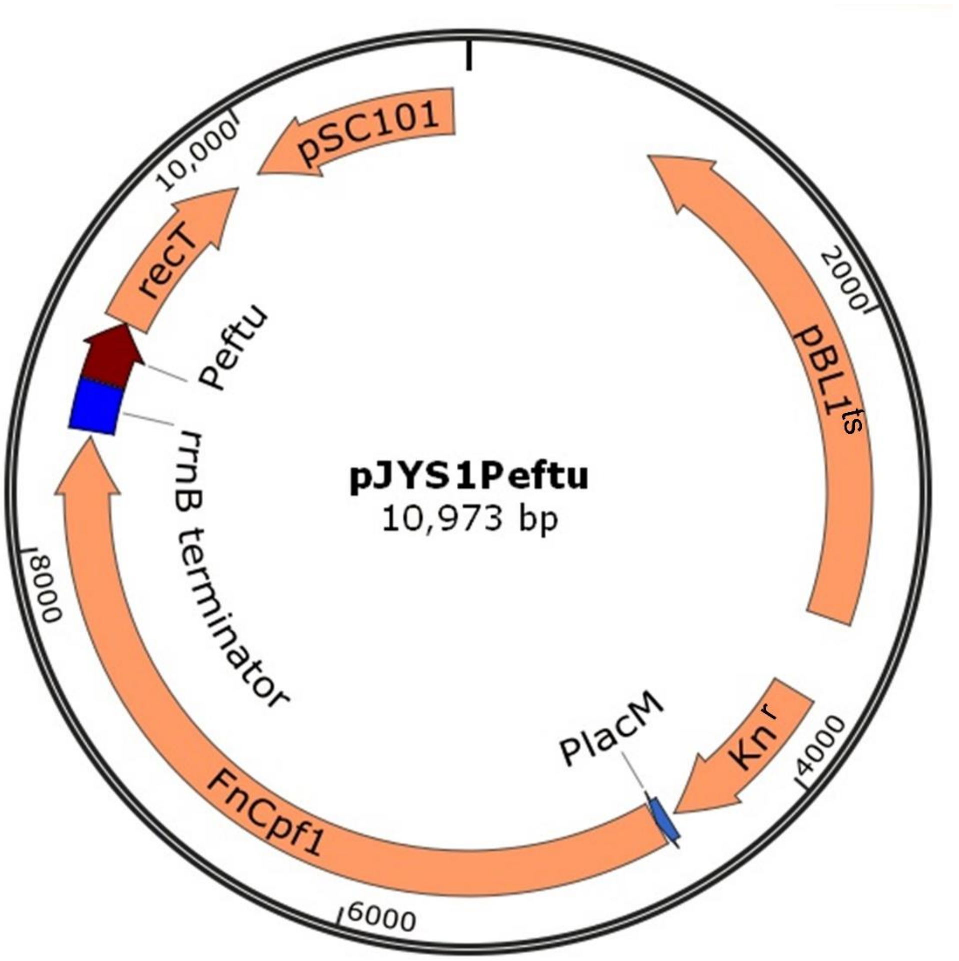

Supplementary Figure 1. Plasmid map: Map of *Fn*Cpf1 expression plasmid pJYS1Peftu of the double-plasmid-based CRISPR-Cpf1 system, including *Fn*Cpf1 and RecT expression modules.

pBL1^{ts}: temperature-sensitive replication derived from the pBL1 replicon of *C. glutamicum*; pSC101: pSC101 replication origin of *E. coli*; *recT*: gene encoding the recombination and repair protein; Kn^{r}: kanamycin resistance gene encoded by the aminoglycoside phosphotransferase gene; PlacM: modified *lac* constitutive expression promoter in *C. glutamicum*; Peftu: the *eftu* promoter of the *tuf* gene in *C. glutamicum*; *Fn*Cpf1: Cpf1 derived from *F. novicida* (NC_008601)

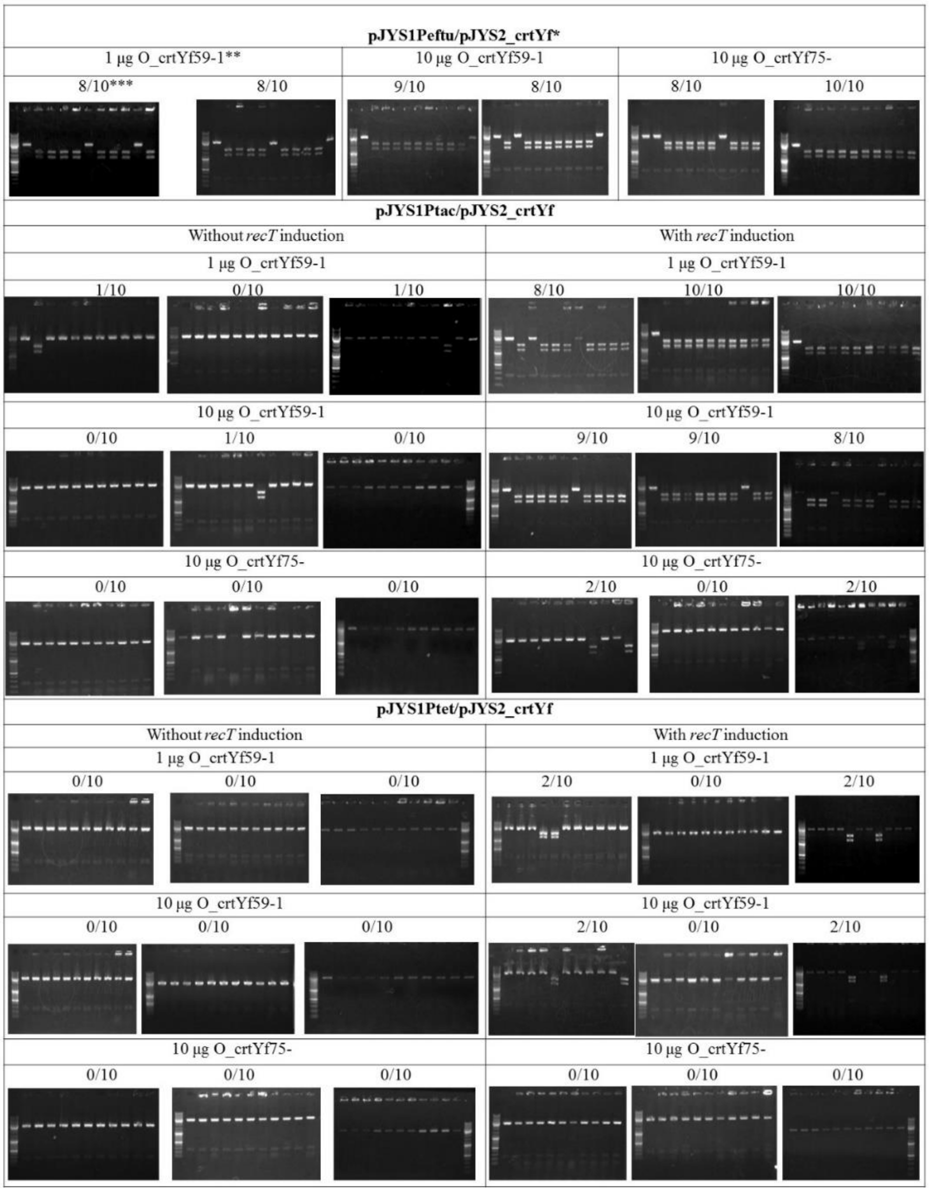

Supplementary Figure 2. PCR validation of colonies obtained by the pJYS1 series/pJYS2_*crtYf*-based CRISPR-Cpf1 recombineering experiments. Ten colonies derived from the oligonucleotide-mediated pJYS1 series/pJYS2_*crtYf*-based CRISPR-Cpf1 recombineering experiment were screened by colony PCR, followed by HpaI digestion, to identify recombinants in the *crtYf* locus as indicated in Figure 2c. Lane 1 (from left), marker; lane 2, wild-type genotype as a negative control; lanes 3-12, samples from the transformants. A 2.9-kb fragment is indicative of the wild-type genotype, whereas the presence of 1.6-kb, 1.1-kb, and 0.2-kb fragments is indicative of recombinant genotypes. *Double plasmids used for CRISPR-Cpf1 recombineering. **oligonucleotide used for CRISPR-Cpf1 recombineering. *** (n/N): n, number of editing-positive transformants; N, number of transformants tested. A DNA ladder (GeneRuler, Thermo Scientific) was used as a marker. *C. glutamicum* ATCC13032 wild-type genomic DNA was used as negative template controls.

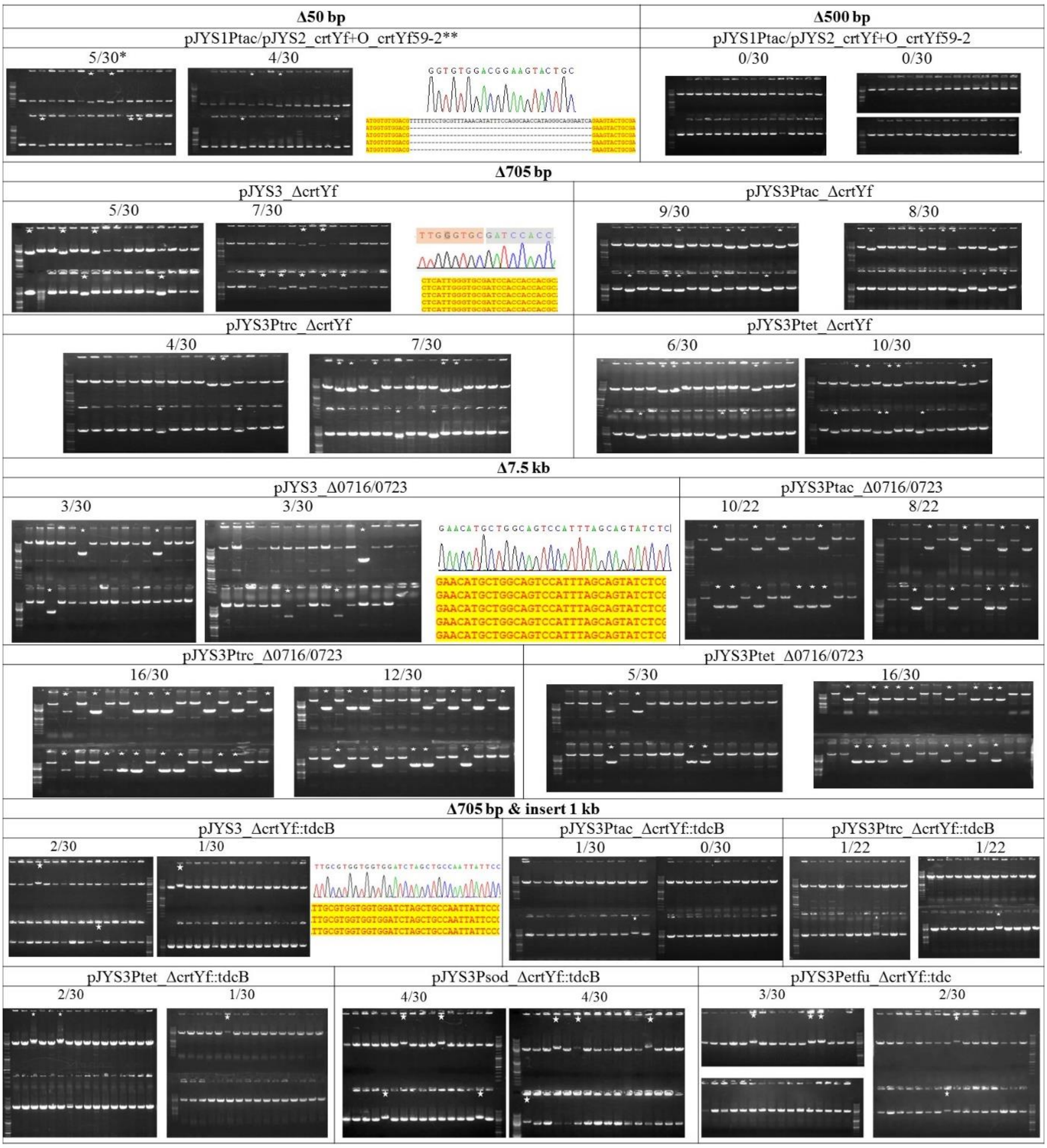

Supplementary Figure 3. PCR validation of colonies obtained by CRISPR-Cpf1-mediated gene deletion and insertion experiments. Colonies derived from genome editing experiments, as indicated in Figure 3a, were screened by colony PCR, and the positive recombinants were further identified by sequencing as necessary. *(n/N): n, number of editing-positive transformants; N, number of transformants tested. A DNA ladder (GeneRuler, Thermo Scientific) was used as a marker. *C. glutamicum* ATCC13032 wild-type genomic DNA was used as negative template controls. For 50 bp, 705 bp, 7.5 kb deletion, and 1 kb insertion case, positive recombinants of 305 bp, 2356 bp, 2.5 kb, and 3.7 kb are marked with stars in agarose gel, while the wild genotype are 355 bp, 3061 bp, 10 kb, and 3.1 kb, respectively.

pJYS1Ptac/pJYS2_*argR*+ O_argR59-1, 324/325AC>CT (Q109AMB)

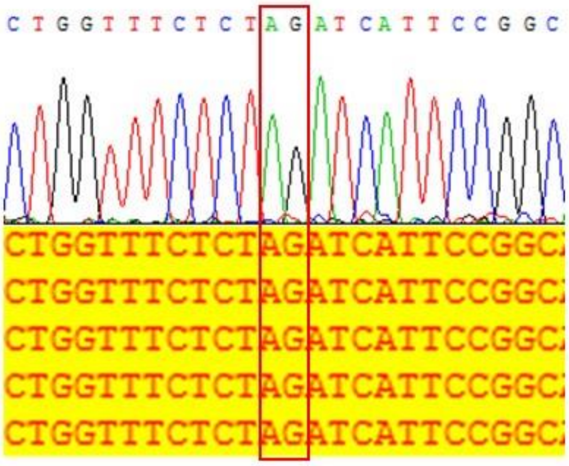

pJYS1Ptac/pJYS2_*argR*+ O_argR59-2, 17-bp deletion

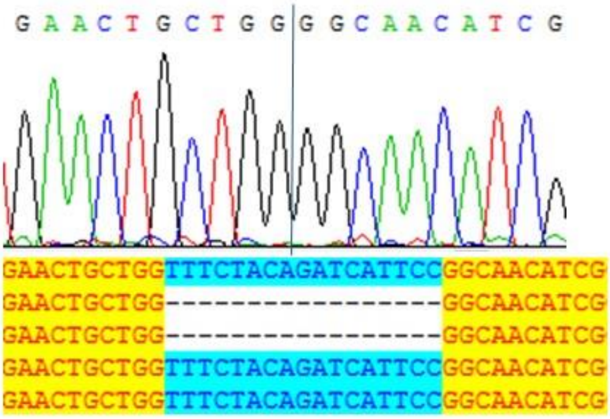

Supplementary Figure 4. Sanger sequence chromatograms of editing sites at *argR*. 5 individual colonies were examined by Sanger sequencing for 324/325AC>CT substitutions or 17-bp deletions at positions of *argR*.

Supplementary Tables

Supplementary Table 1. Twenty oligonucleotides for ProB saturation mutagenesis at codon 149.

Primers	Sequences*
Original sequence	TCGGTCGTTGTCACCAAAATTCACACCGGTGGTTGCCACGGTGTCATTTTCATTGACGA
G149amb	TCGGTCGTTGTCACCAAAATTCAC**T**C**AA**GT**A**GT**A**GCCACGGTGTCATTTTCATTGACGA
G149A	TCGGTCGTTGTCACCAAAATTCAC**TG**C**A**GT**A**GT**A**GCCACGGTGTCATTTTCATTGACGA
G149C	TCGGTCGTTGTCACCAAAATTCAC**G**C**AA**GT**A**GT**A**GCCACGGTGTCATTTTCATTGACGA
G149D	TCGGTCGTTGTCACCAAAATTCACA**T**C**A**GT**A**GT**A**GCCACGGTGTCATTTTCATTGACGA
G149E	TCGGTCGTTGTCACCAAAATTCAC**TT**C**A**GT**A**GT**A**GCCACGGTGTCATTTTCATTGACGA
G149F	TCGGTCGTTGTCACCAAAATTCAC**GAAA**GT**A**GT**A**GCCACGGTGTCATTTTCATTGACGA
G149H	TCGGTCGTTGTCACCAAAATTCAC**GTGA**GT**A**GT**A**GCCACGGTGTCATTTTCATTGACGA
G149I	TCGGTCGTTGTCACCAAAATTCAC**GATA**GT**A**GT**A**GCCACGGTGTCATTTTCATTGACGA
G149K	TCGGTCGTTGTCACCAAAATTCAC**CTTA**GT**A**GT**A**GCCACGGTGTCATTTTCATTGACGA
G149L	TCGGTCGTTGTCACCAAAATTCAC**CAGA**GT**A**GT**A**GCCACGGTGTCATTTTCATTGACGA
G149M	TCGGTCGTTGTCACCAAAATTCAC**CATA**GT**A**GT**A**GCCACGGTGTCATTTTCATTGACGA
G149N	TCGGTCGTTGTCACCAAAATTCAC**GTTA**GT**A**GT**A**GCCACGGTGTCATTTTCATTGACGA
G149P	TCGGTCGTTGTCACCAAAATTCAC**TGGA**GT**A**GT**A**GCCACGGTGTCATTTTCATTGACGA
G149Q	TCGGTCGTTGTCACCAAAATTCAC**CTGA**GT**A**GT**A**GCCACGGTGTCATTTTCATTGACGA
G149R	TCGGTCGTTGTCACCAAAATTCAC**G**C**GA**GT**A**GT**A**GCCACGGTGTCATTTTCATTGACGA
G149S	TCGGTCGTTGTCACCAAAATTCAC**GGAA**GT**A**GT**A**GCCACGGTGTCATTTTCATTGACGA
G149T	TCGGTCGTTGTCACCAAAATTCAC**GGTA**GT**A**GT**A**GCCACGGTGTCATTTTCATTGACGA
G149V	TCGGTCGTTGTCACCAAAATTCAC**CA**C**A**GT**A**GT**A**GCCACGGTGTCATTTTCATTGACGA
G149W	TCGGTCGTTGTCACCAAAATTCAC**C**C**AA**GT**A**GT**A**GCCACGGTGTCATTTTCATTGACGA
G149Y	TCGGTCGTTGTCACCAAAATTCAC**GTAA**GT**A**GT**A**GCCACGGTGTCATTTTCATTGACGA

* The PAM regions are underlined with double lines; codon 149 is shaded in gray; and the substitution nucleotides are indicated in bold.

Supplementary Table 2. List of codons obtained at site 149 of ProB from the saturation mutagenesis library*.

No. of mutants	Codon 149	Amino acid	
WT	GGT	Glycine	G
1	TGA	termination codon	Amb
4	GCA	Alanine	A
3	TGC	Cysteine	C
1	GAT	Aspartic acid	D
2	GAA	Glutamic acid	E
1	CTG	Leucine	L
1	ATG	Methionine	M
2	AAC	Asparagine	N
2	CGC	Arginine	R
1	TCC	Serine	S
1	GTG	Valine	V
5	TGG	Tryptophan	W
1	TAC	Tyrosine	Y

* Thirty colonies were picked randomly for ProB codon 149 sequencing, and 25 showed the expected substitution as listed.

Supplementary Table 3. L-proline fermentation by 190 randomly picked colonies from the ProB G149 saturation mutagenesis library*.

Transformants No.	Proline (g l^{-1})	Transformants No.	Proline (g l^{-1})	Transformants No.	Proline (g l^{-1})
56	7.49±0.73	107	3.42	74	1.55
57	7.40±0.63	140	3.40	40	1.54
72	6.77±0.29	117	3.35	59	1.49
41	5.42±0.74	36	3.34	71	1.45
62	5.47±0.54	152	3.31	64	1.37
61	6.11±0.39	151	3.30	60	1.35
29	4.63±0.94	20	3.30	15	1.30
28	5.22±0.39	14	3.28	105	1.28
53	4.92±0.63	116	3.26	67	1.28
32	5.31±0.41	18	3.26	179	1.24
169	6.03±0.61	136	3.25	83	1.22
70	4.56±0.56	143	3.25	84	1.20
187	5.72±0.52	27	3.21	82	1.16
35	3.79±1.12	104	3.19	159	1.15
90	4.69±0.35	102	3.19	154	1.15
133	6.11±0.97	80	3.18	168	1.14
153	5.25±0.29	130	3.18	25	0.93±0.19
52	3.94±0.94	138	3.15	171	1.13
145	6.23±1.05	87	3.13	86	1.13
68	4.46±0.45	141	3.07	174	1.10
51	3.74±0.95	149	3.01	189	1.09
2	5.93±0.99	127	3.00	95	1.08
38	4.37±0.42	4	2.99	164	1.07
42	3.60±0.96	148	2.98	167	1.07
39	3.90±0.80	8	2.98	122	1.04
34	3.91±0.80	76	2.96	173	1.03
77	3.36±1.12	137	2.86	191	1.03
92	4.63±0.09	109	2.85	155	1.02
135	5.32±0.71	156	2.82	125	1.01
69	4.28±0.18	139	2.80	161	1.01
66	2.85±1.37	121	2.80	111	1.01
75	3.06±1.17	97	2.79	172	1.00
44	4.19±0.36	3	2.67	186	1.00
88	3.02±1.12	184	2.66	89	0.99
16	3.53±0.66	55	2.59	144	0.98
24	3.25±0.88	6	2.58	160	0.98
22	2.90±1.18	157	2.56	128	0.97
79	3.08±1.02	178	2.46	177	0.97
21	3.25±0.78	166	2.25	45	0.96

49	3.56±0.49	142	2.23	123	0.95
23	4.05±0.91	54	2.23	9	0.95
13	3.99±0.06	150	2.22	114	0.94
47	3.97	132	2.22	5	0.91
48	3.96	43	2.19	192	0.91
120	3.95	85	2.16	165	0.90
91	3.93	162	2.13	182	0.90
7	3.88	108	2.07	180	0.89
96	3.78	126	2.05	118	0.88
17	3.75	37	2.03	112	0.86
30	3.73	188	2.03	131	0.86
134	3.73	190	2.02	175	0.86
113	3.73	147	1.82	176	0.85
163	3.70	78	1.80	98	0.85
110	3.69	99	1.78	65	0.85
58	3.68	106	1.78	119	0.83
93	3.65	100	1.71	115	0.82
103	3.57	10	1.65	124	0.81
81	3.54	185	1.64	1	0.97±0.18
26	3.51	19	1.64	183	0.73
129	3.49	31	1.64	181	0.71
63	3.45	50	1.63	170	0.64
12	3.44	94	1.61	146	0.51
101	3.43	73	1.60	158	0.51
11	3.43	46	1.57	33	0.11

* Transformants with L-proline titers >4.0 g L^{-1} in the first fermentation experiment (the 42 strains indicated in blue) were analyzed for fermentation in triplicate and were subjected for *proB* sequencing. Nos. 1 and 192 (red font) are data from the wild-type ATCC13032 as baseline control.

Supplementary Table 4. Sequencing results of codon 149 of ProB from the transformants having L-proline titers >4.0 g L^{-1} as listed in Table S5.

Transformants No.	Proline (g l^{-1})	Codon 149	Amino acid	Characteristics*
1	0.97±0.18	GGT	G	WT
192	0.91	GGT	G	WT
56	7.49±0.73	AAG	K	MT
57	7.40±0.63	ACC	T	MT
72	6.77±0.29	ACC	T	MT
41	5.42±0.74	CGC	R	MT
62	5.47±0.54	CAG	Q	MT
61	6.11±0.39	ACC	T	MT
29	4.63±0.94	AAC	N	MT
28	5.22±0.39	CGC	R	MT
53	4.92±0.63	CAG	Q	MT
32	5.31±0.41	GTG	V	MT
169	6.03±0.61	ACC	T	MT
70	4.56±0.56	CGC	R	MT
187	5.72±0.52	GAT	D	MT
35	3.79±1.12	GCA	A	MT
90	4.69±0.35	CAG	Q	MT
133	6.11±0.97	AAG	K	MT
153	5.25±0.29	GAT	D	MT
52	3.94±0.94	CTG	L	MT
145	6.23±1.05	AAG	K	MT
68	4.46±0.45	AAC	N	MT
51	3.74±0.95	CTG	L	MT
2	5.93±0.99	GAT	D	MT
38	4.37±0.42	CAC	H	MT
42	3.60±0.96	TGG	W	MT
39	3.90±0.80	ATC	I	MT
34	3.91±0.80	TGG	W	MT
77	3.36±1.12	TCC	S	MT
92	4.63±0.09	CGC	R	MT
135	5.32±0.71	ACC	T	MT
69	4.28±0.18	CAG	Q	MT
66	2.85±1.37	TGC	C	MT
75	3.06±1.17	CTG	L	MT
44	4.19±0.36	CAG	Q	MT
88	3.02±1.12	TGC	C	MT
16	3.53±0.66	CGC	R	MT
24	3.25±0.88	TAC	Y	MT
22	2.90±1.18	GCA	A	MT
79	3.08±1.02	GCA	A	MT

21	3.25±0.78	TCC	S	MT
49	3.56±0.49	TCC	S	MT
23	4.05±0.91	ATC	I	MT
13	3.99±0.06	CTG	L	MT

*WT, wild type strain; MT, mutant strain.

Supplementary Table 5. Strains and plasmids used in this study.

Strains and plasmids	Characteristics*	Source/Reference
**Strain**		
E. coli DH5α	*F- eNDA1 glnV44 thi-1 recA1 relA1 gyrA96 deoR nupG Φ80dlacZΔM15 Δ(lacZYA-argF)U169, hsdR17 (rK-mK), λ–*	Takara Biotechnology Co.,Ltd.
C. glutamicum ATCC13032	Type strain	American Type Culture Collection (ATCC)
C. acetoacidophilum B230	*Corynebacterium* strain	Shanghai Industrial Microbiology Institute Tech. Co., Ltd.
C. acetoacidophilum B299	*Corynebacterium* strain	Shanghai Industrial Microbiology Institute Tech. Co., Ltd.
C. glutamicum B1	*Corynebacterium* strain	Shanghai Industrial Microbiology Institute Tech. Co., Ltd.
C. pekinense B3	*Corynebacterium* strain	Shanghai Industrial Microbiology Institute Tech. Co., Ltd.
C. crenatum B6	*Corynebacterium* strain	Shanghai Industrial Microbiology Institute Tech. Co., Ltd.
C. glutamicum B226	*Corynebacterium* strain	Shanghai Industrial Microbiology Institute Tech. Co., Ltd.
**Plasmids**		
pTrc99A	*E. coli* cloning vector; pMB1 $oriV_{E. coli}$ Ap^r *lacIq*	[1]
pMW119	*E. coli* cloning vector; pSC101 $oriV_{E. coli}$ Ap^r *lacZ*	Nippon Gene Co.
pXMJ19	*E. coli–C. glutamicum* shuttle vector; Cm^r Ptac *lacIq* pMB1 $oriV_{E. coli}$ pBL1 $oriV_{C. glutamicum}$	Kindly provided by Professor Shuangjiang Liu (Institute of Microbiology, Chinese Academy of Sciences) [2]
pEKEx2	*E. coli–C. glutamicum* shuttle vector; Kn^r Ptac *lacIq* pUC18 MCS pMB1 $oriV_{E. coli}$ pBL1 $oriV_{C. glutamicum}$	Kindly provided by Professor Shuangjiang Liu (Institute of Microbiology, Chinese Academy of Sciences) [3]
pSenlys-Spec	Encodes LysG, and its target promoter fused to *eyfp*; Sp^r	Kindly provided by Professor Lothar Eggeling (Forschungszentrum Jülich) [4]
pTRCmob	*E. coli–C. glutamicum* shuttle vector; Kn^r Ptac mob pAG1 $oriV_{C. glutamicum}$ pMB1 $oriV_{E. coli}$	Kindly provided by Professor Shuangjiang Liu (Institute of Microbiology, Chinese Academy of Sciences) [5]
pTRCmob_sp	Derived from pTRCmob; Sp^r	This study
pXMJ19ts-Pncas9	Kn^r Ptac *lacIq* $pBL1^{ts}$ $oriV_{C. glutamicum}$ pSC101 $oriV_{E. coli}$ *SpCas9* with native promoter	This study
pXMJ19ts-Plcas9	Derived from pXMJ19ts-Pncas9; PlacM-*Sp*Cas9	This study
pXMJ19ts-Plcas9n	Derived from pXMJ19ts-Pncas9; *Sp*Cas9 nickase (D10A)	This study
pXMJ19ts-Plcpf1	Derived from pXMJ19ts-Pncas9; PlacM-*Fn*Cpf1	This study
pXMJ19ts-Plcpf1n	Derived from pXMJ19ts-Plcpf1; *Fn*Cpf1 (R1218A)	This study
pXMJ19ts-Plcpf1-crRNAcrtYf	Derived from pXMJ19ts-Plcpf1; Pj23119-crRNA targeting *crtYf*	This study
pXMJ19ts-Plcpf1n-crRNAcrtYf	Derived from pXMJ19ts-Plcpf1-crRNAcrtYf; *Fn*Cpf1 (R1218A)	This study
**Double-plasmid-based CRISPR–Cpf1 system: *Fn*Cpf1 expression plasmids**		
pJYS1Peftu	$pBL1^{ts}$ $oriV_{C. glutamicum}$ Kn^r pSC101 $oriV_{E. coli}$ PlacM-*Fn*Cpf1, Peftu-	This study (Addgene: 85546)

	RecT	
pJYS1Ptac	pBL1[ts] *oriV*$_{C.\ glutamicum}$ Kn[r] pSC101 *oriV*$_{E.\ coli}$ *lacIq*, PlacM-*Fn*Cpf1, Ptac-RecT	This study (Addgene: 85545)
pJYS1Ptet	pBL1[ts] *oriV*$_{C.\ glutamicum}$ Kn[r] pSC101 *oriV*$_{E.\ coli}$ *tetR*, PlacM-*Fn*Cpf1, Ptet-RecT	This study
**Double-plasmid-based CRISPR–Cpf1 system: crRNA expression plasmids**		
pJYS2_crtYf	*rep oriV*$_{C.\ glutamicum}$ Sp[r] pMB1 *oriV*$_{E.\ coli}$ Pj23119-crRNA targeting *crtYf*	This study (Addgene: 85544)
pJYS2_argR	*rep oriV*$_{C.\ glutamicum}$ Sp[r], pMB1 *oriV*$_{E.\ coli}$ Pj23119-crRNA targeting *argR*	This study
pJYS2_proB1	*rep oriV*$_{C.\ glutamicum}$ Sp[r] pMB1 *oriV*$_{E.\ coli}$ Pj23119-crRNA targeting *proB*	This study
pJYS2_proB2	*rep oriV*$_{C.\ glutamicum}$ Sp[r], pMB1 *oriV*$_{E.\ coli}$ Pj23119-crRNA targeting *proB*	This study
pJYS2_proB3	*rep oriV*$_{C.\ glutamicum}$ Sp[r] pMB1 *oriV*$_{E.\ coli}$ Pj23119-crRNA targeting *proB*	This study
**All-in-one CRISPR-Cpf1 plasmids**		
pJYS3_ΔcrtYf	pBL1[ts] *oriV*$_{C.\ glutamicum}$ Kn[r] pSC101 *oriV*$_{E.\ coli}$ PlacM-FnCpf1, Pj23119-crRNA targeting *crtYf*, 1-kb upstream and downstream homologous arms flanking 705-bp deletion fragment inside *crtYf*	This study (Addgene: 85542)
pJYS3Ptac_ΔcrtYf	Derived from pJYS3_ΔcrtYf; Ptac-*Fn*Cpf1, *lacIq*	This study
pJYS3Ptrc_ΔcrtYf	Derived from pJYS3_ΔcrtYf; Ptrc-*Fn*Cpf1, *lacIq*	This study
pJYS3Ptet_ΔcrtYf	Derived from pJYS3_ΔcrtYf; Ptet-*Fn*Cpf1, *tetR*	This study
pJYS3_Δcg0716/0723	Derived from pJYS3_ΔcrtYf; 1-kb upstream and downstream homologous region flanking 7.5-kb deletion from *cg0716* to *cg0723*	This study
pJYS3Ptac_Δcg0716/0723	Derived from pJYS3_Δcg0716/0723; Ptac-*Fn*Cpf1, *lacIq*	This study
pJYS3Ptrc_Δcg0716/0723	Derived from pJYS3_Δcg0716/0723; Ptrc-*Fn*Cpf1, *lacIq*	This study
pJYS3Ptet_Δcg0716/0723	Derived from pJYS3_Δcg0716/0723; Ptet-*Fn*Cpf1, *tetR*	This study
pJYS3_ΔcrtYf::tdcB	Derived from pJYS3_ΔcrtYf; Psod-*tdcB* (1.3 kb) inserted between the 1-kb upstream and downstream homologous region flaking the 705-bp deletion fragment inside *crtYf*	This study
pJYS3Psod_ΔcrtYf::tdcB	Derived from pJYS3_ΔcrtY; Psod-*Fn*Cpf1	This study
pJYS3Peftu_ΔcrtYf::tdcB	Derived from pJYS3_ΔcrtY; Petfu-*Fn*Cpf1	This study
pJYS3Ptac_ΔcrtYf::tdcB	Derived from pJYS3_ΔcrtY; Ptac-*Fn*Cpf1, *lacIq*	This study
pJYS3Ptrc_ΔcrtYf::tdcB	Derived from pJYS3_ΔcrtY; Ptrc-*Fn*Cpf1, *lacIq*	This study
pJYS3Ptet_ΔcrtYf::tdcB	Derived from pJYS3_ΔcrtY; Ptet-*Fn*Cpf1, *tetR*	This study
pJYS3Psod_crtYf	Derived from pJYS3Psod_ΔcrtYf::tdcB; homologous region deleted	This study
pJYS3Peftu_crtYf	Derived from pJYS3Peftu_ΔcrtYf::tdcB; homologous region deleted	This study
pJYS3Ptac_crtYf	Derived from pJYS3Ptac_ΔcrtYf; homologous region deleted	This study
pJYS3Ptrc_crtYf	Derived from pJYS3Ptrc_ΔcrtYf; homologous region deleted	This study
pJYS3Ptet_crtYf	Derived from pJYS3Ptet_ΔcrtYf; homologous region deleted	This study

* Ap[r], ampicillin resistance gene encoded by *bla*; Cm[r], chloramphenicol resistance gene encoded by *cat*; Sp[r], spectinomycin resistance gene encoded by *aad9*; Kn[r], kanamycin resistance gene encoded by *aph(3')-IIa*.

Supplementary References

1. Amann, E., Ochs, B. & Abel, K. J. Tightly regulated tac promoter vectors useful for the expression of unfused and fused proteins in *Escherichia coli*. *Gene.* **69**, 301-315 (1988).

2. Shen, X. H., Jiang, C. Y., Huang, Y., Liu, Z. P. & Liu, S. J. Functional identification of novel genes involved in the glutathione-independent gentisate pathway in *Corynebacterium glutamicum*. *Appl Environ Microbiol.* **71**, 3442-3452 (2005).

3. Eikmanns, B. J., Kleinertz, E., Liebl, W. & Sahm, H. A family of Corynebacterium glutamicum/Escherichia coli shuttle vectors for cloning, controlled gene expression, and promoter probing. *Gene.* **102**, 93-98 (1991).

4. Schendzielorz, G. *et al.* Taking control over control: use of product sensing in single cells to remove flux control at key enzymes in biosynthesis pathways. *ACS Synth Biol.* **3**, 21-29 (2014).

5. Liu, Q., Ouyang, S. J., Kim, J. & Chen, G. Q. The impact of PHB accumulation on L-glutamate production by recombinant *Corynebacterium glutamicum*. *J Biotechnol.* **132**, 273-279 (2007).
